# Waxberry‐Shaped Ordered Mesoporous P‐TiO_2−*x*_ Microspheres as High‐Performance Cathodes for Lithium–Sulfur Batteries

**DOI:** 10.1002/smsc.202200032

**Published:** 2022-12-01

**Authors:** Wenna Zhang, Yuanmei Xu, Jiabing Liu, Yebao Li, Eser Metin Akinoglu, Yaojie Zhu, Yongguang Zhang, Xin Wang, Zhongwei Chen

**Affiliations:** ^1^ South China Academy of Advanced Optoelectronics International Academy of Optoelectronics at Zhaoqing South China Normal University Guangdong 510006 China; ^2^ School of Materials Science and Engineering Hebei University of Technology Tianjin 300130 China; ^3^ Department of Chemical Engineering University of Waterloo Waterloo ON N2L 3G1 Canada

**Keywords:** lithium–sulfur batteries, phosphorus doping, radial mesoporous structures, waxberry‐shaped titanium dioxide

## Abstract

As a candidate for a new generation of inexpensive and high‐performance energy storage systems, lithium–sulfur (Li–S) batteries have attracted widespread research. However, the development and application of Li–S batteries are limited by severe polysulfide dissolution and slow reaction kinetics. Herein, a type of ordered mesoporous P‐TiO_2−*x*_ microsphere with a waxberry‐like shape as the sulfur host material for Li–S batteries is put forward, which combines the radially arranged mesoporous structure with oxygen defects in the mesoporous framework. In addition, the introduction of phosphorus impurities greatly improves the conductivity of the sulfur electrode, enhances electron mobility, and promotes the interaction between the sulfur species and P‐TiO_2−*x*_ microspheres. Finally, S/P‐TiO_2−*x*_ cathodes have achieved a high capacity of 1174.9 mAh g^−1^ at 0.2C and stable cycling (the average capacity attenuation is only 0.086% per cycle at 1C after 600 cycles).

## Graphical abstract

An ordered mesoporous P‐doped defective TiO_2_ (P‐TiO_2−*x*_) microsphere with a waxberry‐like shape is prepared as a sulfur host for Li–S batteries. The doping of phosphorus and the introduction of oxygen defects promote charge transfer and enhance the interaction between sulfur species and P‐TiO_2−*x*_. Therefore, the Li–S battery with S/P‐TiO_2−*x*_ cathode shows excellent electrochemical performance.

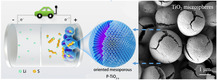

## 1 Introduction

With the rapid development of portable electronic devices and electric vehicles, there is an urgent need to develop a new generation of high‐energy‐density, environmentally friendly, safe, and low‐cost battery systems.^[^ ^1^, ^2^, ^3^ ^]^ Lithium–sulfur (Li–S) batteries are a new type of secondary battery that uses metal lithium as the negative electrode and use sulfur as the positive electrode active material.^[^ ^4^, ^5^ ^]^ Benefiting from the multielectron reaction of sulfur phase change, Li–S batteries have theoretical specific capacities (1675 mAh g^−1^) and specific energy (2600 Wh kg^−1^), which are equivalent to several times that of commercial lithium‐ion batteries.^[^ ^6^, ^7^ ^]^ In addition, with abundant sulfur reserves, low price, and environmentally friendly, Li–S batteries are considered to be the next‐generation high‐energy battery system with the most development potential and application prospects.^[^ ^8^, ^9^ ^]^

Compared with traditional lithium‐ion batteries, the multielectron reaction characteristics of sulfur phase change not only endow Li–S batteries with high specific capacity and specific energy but also lead to several problems in the Li–S battery system: 1) active sulfur and final discharge products electronic and ion insulation, leading to high internal resistance, serious polarization, and low active material utilization in the battery system:^[^ ^10^, ^11^ ^]^ 2) the dissolution of soluble polysulfide and the intermediate product of charge and discharge;^[^ ^12^ ^]^ 3) the electrode volume expansion/shrinkage causes the active material to fall off and the electrode structure is destroyed, which seriously threatens the battery's cycle stability and energy density performance.^[^ ^13^, ^14^, ^15^ ^]^

To overcome the abovementioned obstacles, scientific researchers set out to conduct in‐depth research on the host cathode material of sulfur. Carbon materials (such as carbon nanofibers, carbon nanotubes, and graphene) have been widely used in Li–S batteries.^[^ ^16^, ^17^, ^18^ ^]^ However, the physical interaction between carbon materials and polysulfides is relatively weak, and slow redox kinetics and strong charge transfer resistance will seriously affect battery performance.^[^ ^19^, ^20^ ^]^ Polar metal compounds including metal oxides, nitrides, sulfides, phosphates, and carbides have been shown to have strong chemisorption and catalytic conversion to lithium polysulfides. These polar metal compounds have incomparable advantages over other materials in the conversion process of high‐order lithium sulfide in the second stage to low‐order lithium sulfide, and they are also effective means to inhibit the “shuttle effect” by promoting the transformation of the intermediate product of lithium sulfides to the final product of lithium sulfides.^[^ ^21^ ^]^ Various transition metal oxides have large electrochemically active surfaces containing hydrophilic groups and are expected to serve as host hosts for sulfur.^[^ ^22^ ^]^ However, the low inherent electronic conductivity of metal oxides is a key problem that must be solved.^[^ ^23^ ^]^ Black titanium oxide nanomaterial (TiO_2−*x*_) is a new type of semiconductor used in the development of electronic devices.^[^ ^24^ ^]^ The lack of oxygen of the black TiO_2−*x*_ structure shifts its electronic bandgap to about 1.2–1.7 eV and effectively enhances its photo‐electrochemical activity.^[^ ^25^, ^26^ ^]^ In addition, nonmetal doping can significantly increase the capacitance of black TiO_2−*x*_, thereby improving the charge transfer between the electrolyte/TiO_2−*x*_/current collector.^[^ ^27^, ^28^ ^]^

Herein, we propose a novel ordered mesoporous defect‐induced phosphorus‐doped TiO_2_ nanoparticle (P‐TiO_2−*x*_) microsphere with a waxberry‐like shape. The microspheres have a radial mesoporous arrangement and stable oxygen vacancies in the scaffold, and they have uniform cracks through the control of the morphology. Through the introduction of the appropriate concentration of phosphorus doping, the conductivity of the sulfur electrode is greatly increased, the electron mobility is enhanced, and the interaction between the sulfur and the P‐TiO_2−*x*_ surface is promoted. The S/P‐TiO_2−*x*_ microspheres are used as the cathode materials of the Li–S batteries, showing excellent reversible capacity, rate performance, and excellent cyclability (the average capacity attenuation is only 0.086% per cycle at 1 C after 600 cycles).

## 2 Results and Discussion

**Figure 1:**
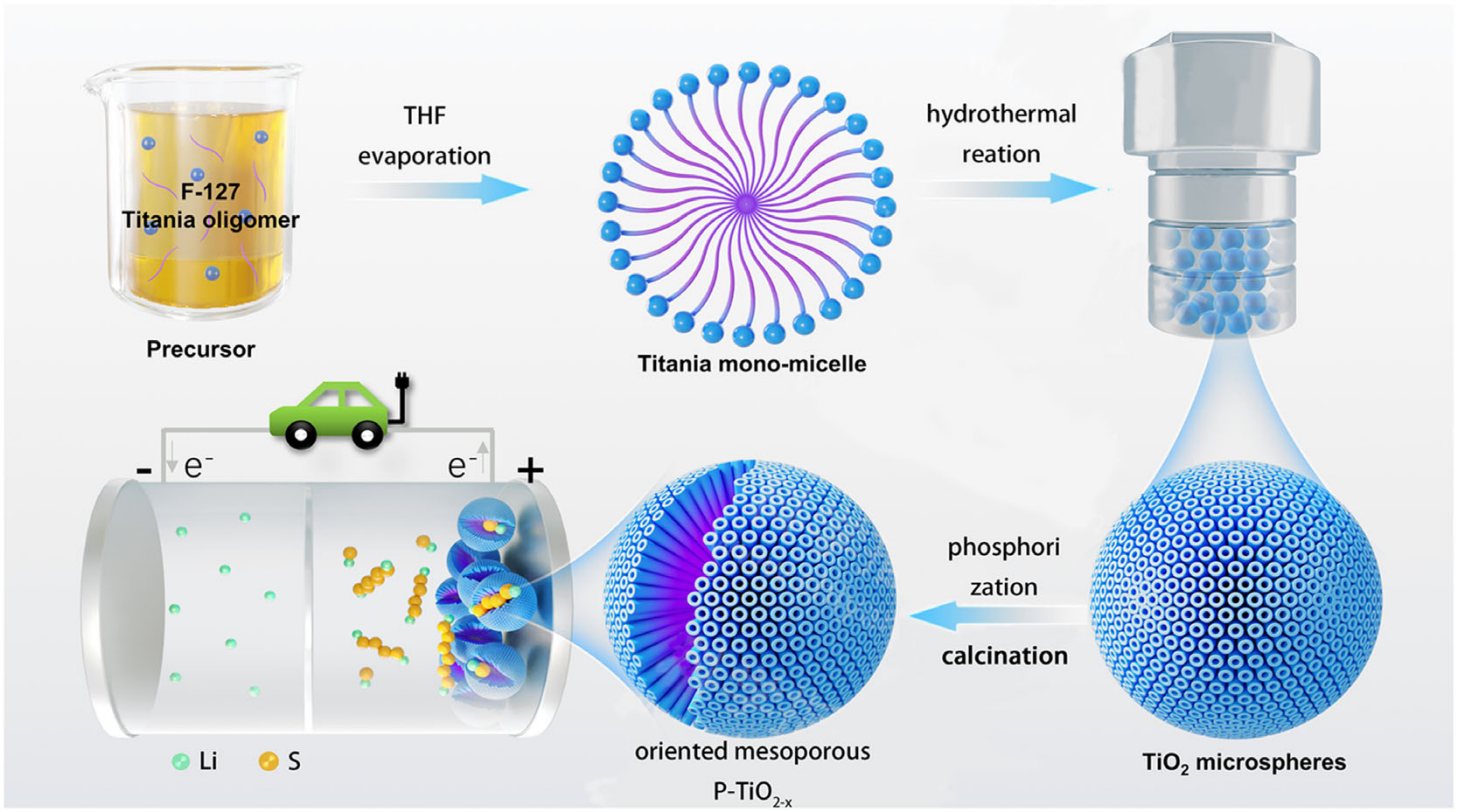
Schematic diagram of the synthesis process for P‐TiO_2−*x*_ microspheres.

The formation mechanism of P‐TiO_2−*x*_ materials is shown in **Figure** **1**. First, TiO_2_ microspheres were synthesized by a simple hydrothermal method. Second, NaBH_4_ and TiO_2_ microspheres were uniformly mixed and heated in an inert atmosphere to form black TiO_2−*x*_ microspheres with a large amount of oxygen defects and Ti^3+^, and the reason for crack formation is that during the hydrothermal process, with continuous hydrolysis and condensation, the amount of *n*‐butanol and water increases, leading to the increase of internal vapor pressure. When the internal vapor pressure exceeds the external hydrothermal pressure, the internal solvent will evaporate from the partial area, causing cracks in the microspheres. In this study, sulfur was incorporated into the P‐TiO_2−*x*_ microspheres by the classic melt diffusion method to obtain S/P‐TiO_2−*x*_ composites. To compare the electrochemical properties, S/TiO_2_ composites were prepared in the same way.

**Figure 2:**
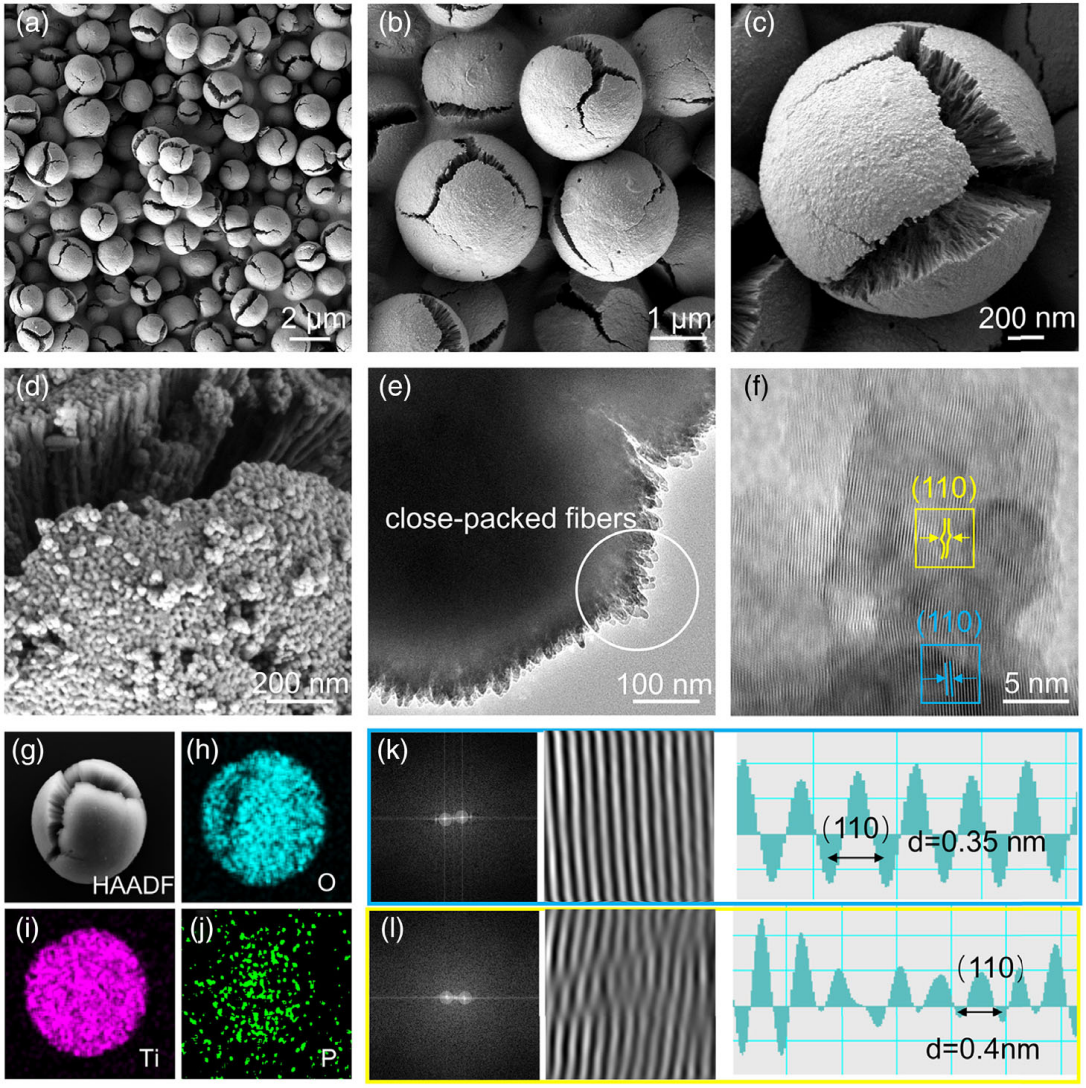
a–d) SEM images of TiO_2_ microspheres (a–c) and P‐TiO_2−*x*_ microspheres (d). e,f) TEM images of P‐TiO_2−*x*_ microspheres. g–j) Corresponding elemental mapping of P‐TiO_2−*x*_ microspheres. k,l) FFT pattern, inverse FFT pattern, and lattice spacing images in the selected area.

SEM images with various magnifications show monodispersed spherical shapes with diameters of about 2.0–3.0 μm, and all microspheres have cracks (**Figure** **2**a–c). Mesoporous channels arranged radially outward from the center can be observed in the P‐TiO_2−*x*_ framework, and cylindrical P‐TiO_2−*x*_ nanocrystals with a width of about 16 nm are formed (Figure 2d), which are further verified by transmission electron microscopy (TEM) imaging (Figure 2e). After phosphating treatment, the surface of P‐TiO_2−*x*_ samples became rough, indicating that the surface had been substantially reconstructed (Figure 2a,d). Figure 2e–l shows the TEM images of P‐TiO_2−*x*_ microspheres and the distribution of related elements. From the P element mapping shown in Figure 2g–j and S1, Supporting Information, we can see the uniform distribution of P elements on P‐TiO_2−*x*_. Furthermore, HAADF‐STEM images of P‐TiO_2−*x*_ microspheres are shown in Figure S2, Supporting Information, P element can be observed, indicating P was doped in TiO_2_. To analyze the crystallinity of P‐TiO_2−*x*_ samples and the existence of oxygen defects, fast Fourier transform (FFT) was used for the selected areas in the blue box and the yellow box for further exploration. We confirmed that the distance between the lattice fringes perpendicular to the radial channel is 3.5 Å and that the plane spacing of the grid corresponds to the (110) atomic plane in rutile P‐TiO_2−*x*_. The FFT result shows that the crystal plane arrangement in the yellow area is damaged (Figure 2k,l). In addition, some dislocations can be seen in the inverse FFT results, confirming the existence of oxygen vacancies in P‐TiO_2−*x*_ samples.^[^ ^29^ ^]^ The TEM images of TiO_2_ microspheres are shown in Figure S3, Supporting Information, no obvious lattice distortion is observed in the high‐resolution images (Figure S3e,f, Supporting Information). The crystal lattice in the blue region is well arranged, which proves that the P‐TiO_2−*x*_ samples still maintain good crystallinity after a small amount of phosphorus doping (about 3.03%, Table S1, Supporting Information). Furthermore, to research the morphology of the P‐TiO_2−*x*_ after sulfur loading, the morphology of S/P‐TiO_2−*x*_ is shown in Figure S4, Supporting Information. It can be found from the TEM and SEM images that the P‐TiO_2−*x*_ still maintains the same morphology as the original. In addition, sulfur is uniformly loaded into P‐TiO_2−*x*_ as evidenced by the element mapping (Figure S4d, Supporting Information).

**Figure 3:**
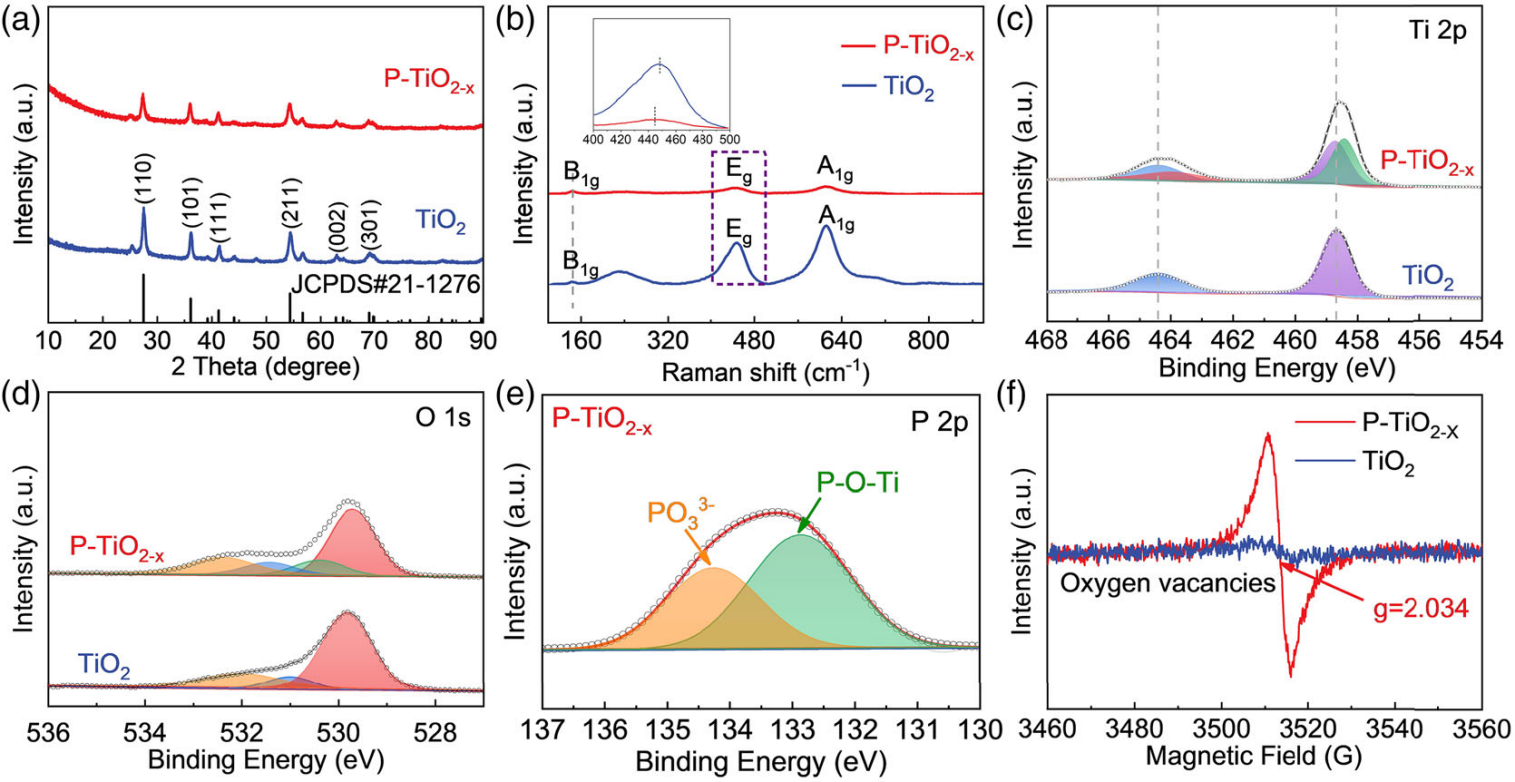
a) XRD patterns of TiO_2_ and P‐TiO_2−*x*_ samples. b) Raman spectra of TiO_2_ and P‐TiO_2−*x*_ samples. c) Ti 2*p* and d) O 1*s* high‐resolution XPS spectra of TiO_2_ and P‐TiO_2−*x*_ microspheres. e) P 2*p* high‐resolution XPS spectra of P‐TiO_2−*x*_ microspheres. f) EPR spectra of TiO_2_ and P‐TiO_2−*x*_ microspheres.

**Figure** **3**a shows the XRD spectra of TiO_2_ microspheres (blue line) and P‐TiO_2−*x*_ microspheres (red line) samples. XRD patterns show characteristic diffraction peaks at 27.6°, 36.2°, 41.5°, 54.4°, and 62.9° which can be indexed as rutile (110), (101), (111), (211), (002) reflections (space group *P*42/*mm*). This means that the rutile phase of TiO_2_ microspheres crystallizes well,^[^ ^30^ ^]^ and a weak diffraction peak at 25.3° can be indexed as the (101) crystal plane of anatase TiO_2_ (JCPDS No. 21‐1272). It can be seen that after reduction and phosphating treatment, the crystalline phase of P‐TiO_2−*x*_ still exists. However, the diffraction intensity of P‐TiO_2−*x*_ microspheres is reduced and the peak is slightly wider, which is due to the doping of P inhibiting the crystallization of TiO_2_ to a certain extent. In addition, there is no obvious shift observed in the XRD pattern of P‐TiO_2−*x*_ compared with TiO_2_, which may be related to the doping amount of P.^[^ ^31^, ^32^ ^]^ The color of the samples also changed from white at the beginning to dark black (Figure S5, Supporting Information). The Raman spectra of TiO_2_ and P‐TiO_2−*x*_ are shown in Figure 3b. The bands at 144.6, 447.2, and 611.5 cm^−1^ correspond to the B_1g_, E_g_, and A_1g_, respectively. Notably, the peak of P‐TiO_2−*x*_ is wider and the intensity is weaker compared with TiO_2_. In addition, the shift of E_g_ signal can be observed, indicating the reduction of Ti–O bond symmetry, which is caused by crystal structure deformation and the introduction of defects.

To determine the various functions of the matrix material, the detailed characteristics of the matrix and the sulfur cathode structure were further explored through a series of characterizations. Brunauer–Emmett–Teller (BET) test was performed on the two materials (Figure S6, Supporting Information), and their nitrogen adsorption/desorption isotherms are all shown as type IV isotherms.^[^ ^33^ ^]^ The specific surface area and the pore volume of P‐TiO_2−*x*_ are 94.68 m^2^ g^−1^ and 0.07 cm^3^ g^−1^, respectively (Table S2, Supporting Information). This indicates that reduction and phosphating treatments can increase the specific surface area.

The existence of P, Ti, and O in P‐TiO_2−*x*_ microspheres was studied by measuring XPS. According to the survey spectrum (Figure S7, Supporting Information), the P signal was only observed in the P‐TiO_2−*x*_ microspheres, which indicates that P‐TiO_2−*x*_ microspheres contain the element P. Ti 2*p* and O 1*s* peaks of two samples were also measured at high resolution (Figure 3c,d). The two peaks at 458.2 and 463.6 eV for P‐TiO_2−*x*_ microspheres could be attributed to Ti^3+^. For the O 1*s* XPS shown in Figure 3d, the O_3_ peak at 532.6 eV can be signed to the presence of oxygen defects.^[^ ^34^, ^35^ ^]^ In addition, the peaks of Ti 2*p* and O 1*s* for P‐TiO_2−*x*_ shift to lower binding energy (BE) compared with those of TiO_2_, which further proves the introduction of O defect and P in P‐TiO_2−*x*_.^[^ ^36^ ^]^ As shown in the P 2*p* XPS spectrum, the two peaks at 132.8 and 134.4 eV could be assigned to the P–O–Ti bond and the PO_3_^3−^ (Figure 3e).^[^ ^36^, ^37^ ^]^ At the same time, the defect properties of the EPR were researched. The obvious signal of P‐TiO_2−*x*_ near *g* = 2.034 could be attributed to the oxygen vacancies (Figure 3f), which further confirms the successful introduction of oxygen vacancies during the phosphorus doping.^[^ ^38^ ^]^

**Figure 4:**
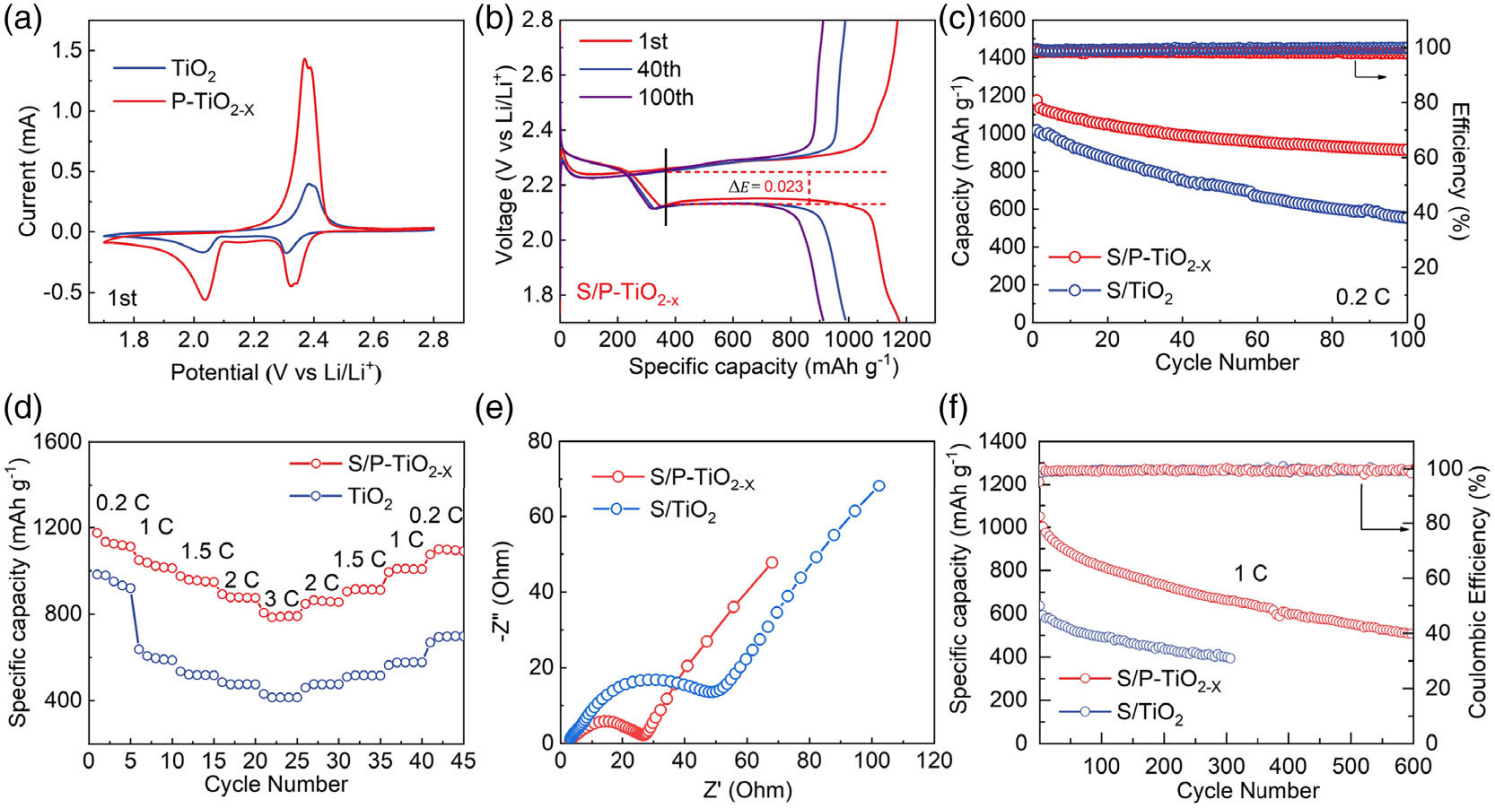
a) CV curves of S/TiO_2_ and S/P‐TiO_2−*x*_. b) Charge/discharge profiles of S/P‐TiO_2−*x*_ electrode at 0.2C. c) Cycling stability at 0.2C. d) Rate capability. e) EIS of the S/TiO_2_ and S/P‐TiO_2−*x*_ cathodes. f) Cycling stability of S/TiO2 and S/P‐TiO_2−*x*_ electrodes at 1C.

**Figure 5:**
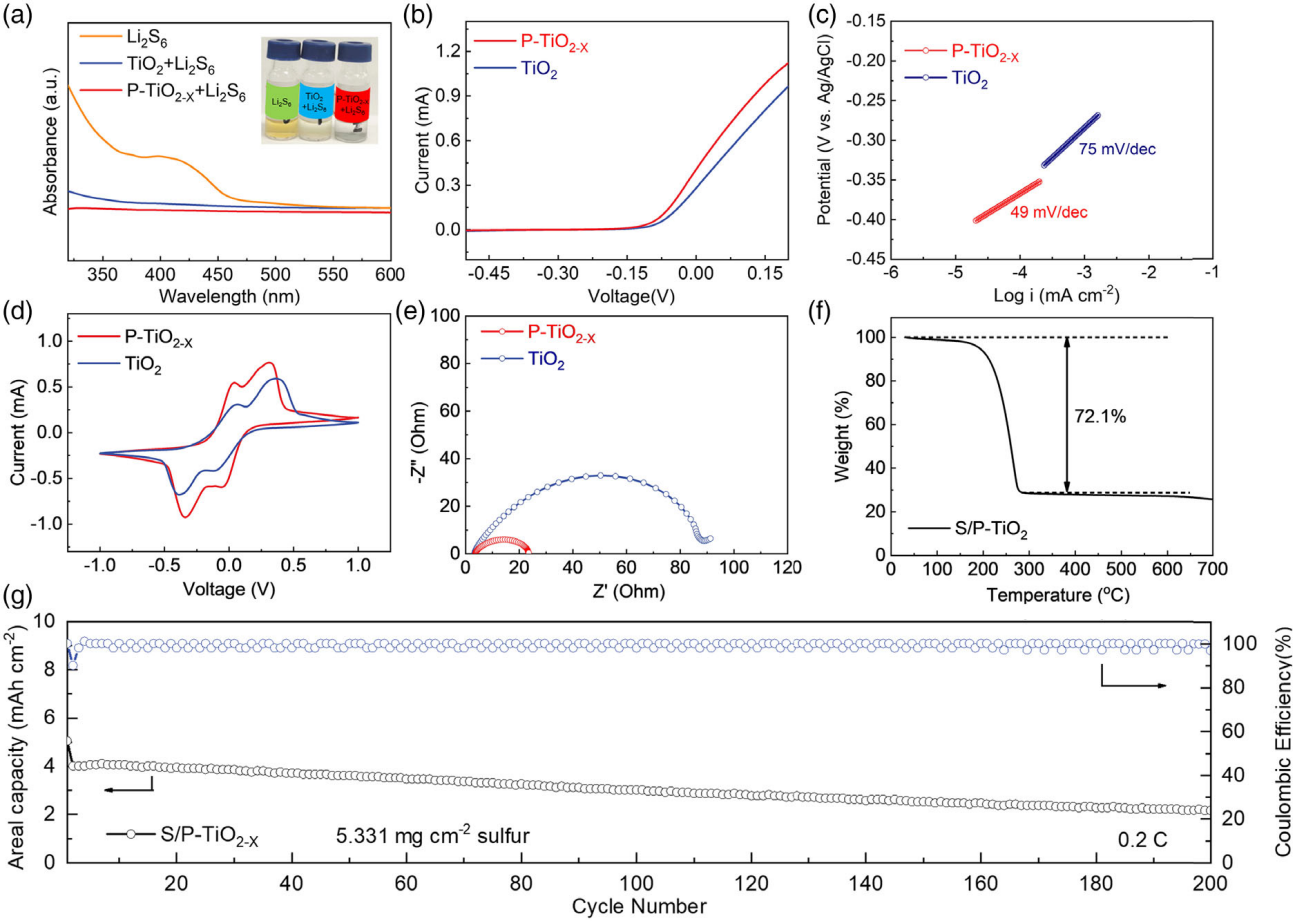
a) Photographs of color contrast and UV–vis spectra of Li_2_S_6_ solution after adsorbed by TiO_2_ and P‐TiO_2−*x*_. b,c) LSV curves and Tafel curve of the S/TiO_2_ and S/P‐TiO_2−*x*_. d,e) CV curves and EIS curves of symmetric cells. f) TGA curve of the S/P‐TiO_2−*x*_. g) High‐loading performances of S/P‐TiO_2−*x*_ cathodes.

**Figure 6:**
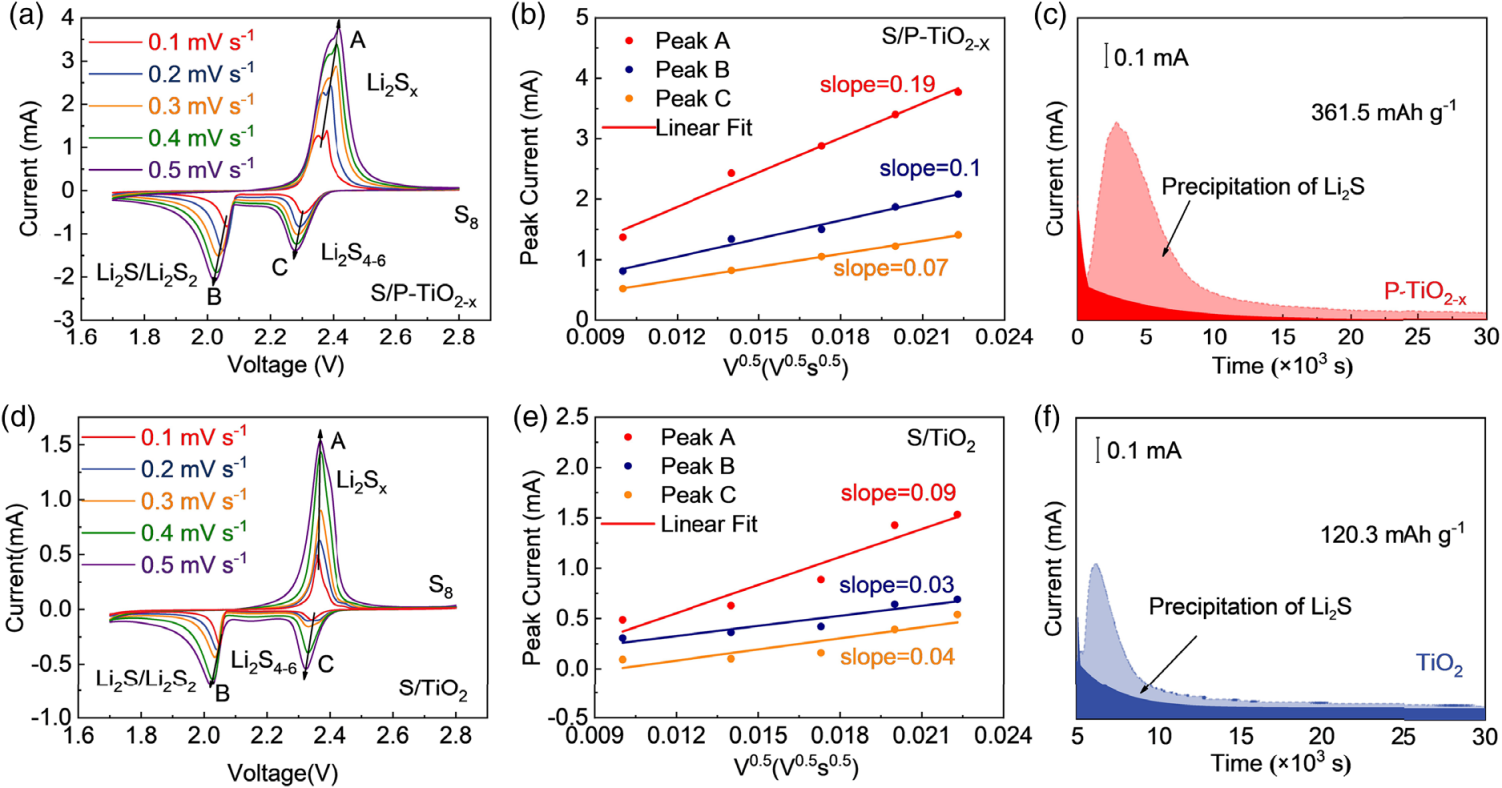
a,d) CV curves at various scan rates for S/P‐TiO_2−*x*_ (a) and S/TiO_2_ (d). b,e) Points and fitted lines of CV peak current versus square root scan rates for S/P‐TiO_2−*x*_ (b) and S/TiO_2_ (e). c,f) Li_2_S deposition test of P‐TiO_2−*x*_ (c) and TiO_2_ (f).

To compare and analyze the effects of phosphorus doping and defect introduction in improving the electrochemical performance of the material, the S/TiO_2_ electrode was used as a comparison. The sulfur composite material is prepared by the melt diffusion method, and the sulfur loading is controlled at about 72.1 wt% (Figure 5f). **Figure** **4**a and 6a,b show the cyclic voltammetry curve of the S/P‐TiO_2−*x*_ electrode and S/TiO_2_ electrode. The S/P‐TiO_2−*x*_ electrode shows significant reduction peaks at 2.32 and 2.03 V, respectively corresponding to the long‐chain lithium polysulfide (Li_2_S_*n*_, 4 ≤ *n* ≤ 8) which is reduced by S_8_ and the reduction of long‐chain lithium polysulfide to short chain Li_2_S_2_ and Li_2_S.^[^ ^39^ ^]^ The battery was then charged, and an oxidation peak appeared around 2.36 V indicating that Li_2_S was oxidized to long‐chain LiPSs, which were further oxidized to elemental S_8_. Compared with S/TiO_2_ materials, S/P‐TiO_2−*x*_ materials show higher peak currents and smaller potential differences (Figure 6d,e). The electrochemical activity of the batteries is improved due to the introduction of phosphorus doping and oxygen defects.

Figure 4b shows the charge and discharge curve of the S/P‐TiO_2−*x*_ electrode under different cycles of 0.2C (1C = 1675 mA g^−1^). As can be seen from the figure, the hysteresis rate (the voltage difference between the oxidation platform and the reduction platform) between the charge and discharge platforms increases gradually as the number of cycles increases. Even after 100 cycles, two discharge platforms in the discharge curve of S/P‐TiO_2−*x*_ electrode are still very obvious, which further confirms the excellent sulfur conversion kinetics in S/P‐TiO_2−*x*_ electrode. As shown in Figure 4c, the S/P‐TiO_2−*x*_ electrode still retains a capacity of 914.2 mAh g^−1^ after 100 cycles at 0.2C, and the capacity retention rate is 77.8%, indicating its excellent cycling performance. The capacitance stability of S/TiO_2_ electrode is obviously lower than that of S/P‐TiO_2−*x*_ electrode. These results indicate that phosphorus doping and the introduction of defects can inhibit the shuttle effect of polysulfides. As shown in Figure 4d, the specific capacity of 1174.9, 1049.8, 975.1, 892.6, and 806.4 mAh g^−1^ is provided at 0.2C, 1C, 1.5C, 2C, and 3C. When the current density is restored to 0.2C, the discharge‐specific capacity can still reach 1,075.6 mAh g^−1^, which may benefit from its higher sulfur utilization rate. We also explored the cycle performance of the material. Even compared with other TiO_2_‐based cathodes for Li–S batteries, the electrochemical performance of S/P‐TiO_2−*x*_ is still competitive as shown in Table S3, Supporting Information.

We also conducted a high‐rate long‐cycle test as shown in Figure 4f to further verify the excellent cycle performance of S/P‐TiO_2−*x*_ materials. The initial discharge‐specific capacity of S/P‐TiO_2−*x*_ electrode at a rate of 1C is as high as 1,049.8 mAh g^−1^, and the average capacity decay rate per cycle is only 0.086% after 600 cycles. In contrast, the initial discharge‐specific capacity of the S/TiO_2_ electrode at 1C is only 637.2 mAh g^−1^. After 300 cycles, the capacity quickly decays to 393.6 mAh g^−1^, and the capacity retention rate is only 61.7%. The previous results indicate that P‐TiO_2−*x*_ materials can effectively inhibit the shuttle effect and have good structural stability.

Figure 4e show the EIS of the S/TiO_2_ electrode and the S/P‐TiO_2−*x*_ electrode before cycling consisting of a semicircle in the high‐frequency region and a straight line in the low‐frequency region. It can be seen that the resistance of S/P‐TiO_2−*x*_ is significantly lower than that of S/TiO_2_, which further shows that the introduction of phosphorus doping and oxygen defects can significantly increase the conductivity of the material, reduce the charge transfer resistance, and provide an effective path for ion and electron transport. To explore the morphology of the P‐TiO_2−*x*_ after cycling, the SEM images of the electrodes after cycling are performed in Figure S8, Supporting Information. Apparently, the structure of P‐TiO_2−*x*_ was not damaged after cycling and maintained a completely spherical shape, indicating that it can effectively adapt to the volume expansion of sulfur during cycling.

To further verify the adsorption capacity of P‐TiO_2−*x*_ and TiO_2_ for LiPSs, the previous samples were dispersed in Li_2_S_6_ solution with a concentration of 5 mM. As shown in **Figure** **5**a, the color of the blank Li_2_S_6_ solution is brown. After P‐TiO_2−*x*_ and TiO_2_ were added to the Li_2_S_6_ solution, the color of the solution became lighter. However, the Li_2_S_6_ solution soaked with P‐TiO_2−*x*_ microspheres is close to colorless, while the color of the Li_2_S_6_ solution soaked with TiO_2_ microspheres is still brownish yellow. It can be seen from the UV–vis spectrum that the characteristic absorption peak of Li_2_S_6_ solution containing P‐TiO_2−*x*_ has almost completely disappeared, which indicates that P‐TiO_2−*x*_ has a strong chemical adsorption capacity for Li_2_S_6_, which is very beneficial to inhibit the shuttle effect and promote the subsequent conversion of LiPSs.^[^ ^40^ ^]^ Moreover, to further explore the interaction between the sulfur species and P‐TiO_2−*x*_, the XPS tests of the Li_2_S_6_ and Li_2_S_6_/P‐TiO_2−*x*_ were performed as shown in Figure S9, Supporting Information. In the S 2*p* spectrum, the peaks of Li_2_S_6_/P‐TiO_2−*x*_ shift to higher BE compared to those of pure Li_2_S_6_, indicating that the electron cloud density of S atoms decreases, and confirming the strong interaction between P‐TiO_2−*x*_ and sulfur species.

To further study the catalytic effect of P‐TiO_2−*x*_ and TiO_2_ on the redox of Li_2_S_6_, the P‐TiO_2−*x*_ electrode sheet (or TiO_2_ electrode sheet) and carbon cloth as the working electrode and counter electrode were used to assemble a symmetrical battery. As shown in Figure 5d, the redox characteristic peaks of the P‐TiO_2−*x*_ symmetric battery are more obvious, indicating that the LiPSs conversion is more sufficient and the conversion efficiency is higher. As shown in Figure 5e, the EIS curve of the P‐TiO_2−*x*_ symmetric battery shows that the charge transfer resistance is less than that of the TiO_2_ battery, which means that the mass transfer resistance of P‐TiO_2−*x*_ boundary is smaller and can promote the rapid conversion of polysulfides. The good effects of P‐TiO_2−*x*_ are further demonstrated in the cases of high sulfur loading cathode. As shown in Figure 5g, at a sulfur content of 5.331 mg cm^−2^, the P‐TiO_2−*x*_ cathode can maintain a 2.2 mAh cm^−2^ areal capacity at 0.2C after 200 cycles. Through linear sweep voltammetry (LSV) and the corresponding Tafel curve, the catalytic conversion effect of different sulfur cathode support materials on LiPSs can be effectively analyzed. As shown in Figure 5b,c, the S/P‐TiO_2−*x*_ electrode exhibits a higher current density and a smaller Tafel slope during oxidation and reduction processes. This indicates that P‐TiO_2−*x*_ material could significantly promote the conversion between LiPSs and Li_2_S/Li_2_S_2_ and exhibit a strong catalytic effect on LiPSs. The previous experimental results show that the presence of phosphorus doping and oxygen defects enhance the catalytic conversion ability of the material to LiPSs, thereby improving the utilization rate of sulfur, making it exhibit good cycle stability and excellent rate performance.

Three‐quarters of the capacity of Li–S batteries comes from the process of discharging and converting Li_2_S_4_ intermediates into Li_2_S. The deposition rate of active materials on the surface of the material is one of the important indicators for studying the reaction kinetics of Li–S batteries. To further study the catalytic effect of P‐TiO_2−*x*_ material, Li_2_S deposition experiments were carried out on the surface of P‐TiO_2−*x*_ and TiO_2_, respectively. The darkened shadow on the left is Li_2_S_8_, which is restored to Li_2_S_6_. While the darkened shadow below is Li_2_S_2_ converted to Li_2_S (**Figure** **6**c,f). Obviously, the P‐TiO_2−*x*_ exhibits a larger capacity of 361.5 mAh g^−1^ than that of TiO_2_ (120.3 mAh g^−1^), demonstrating the outstanding reduction ability of P‐TiO_2−*x*_ to Li_2_S_8_.

**Figure 7:**
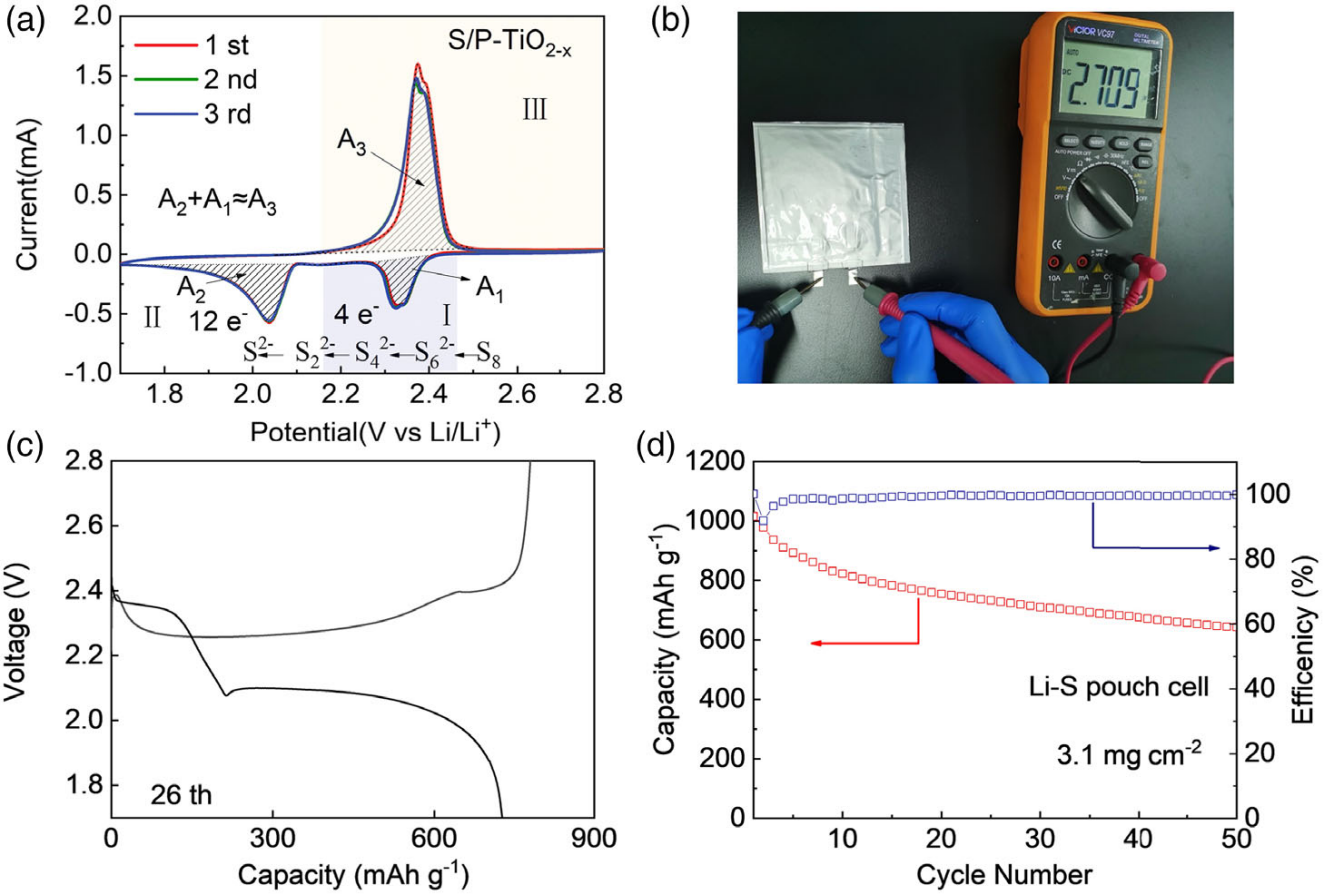
a) CV curves of S/P‐TiO_2−*x*_. b–d) Galvanostatic cycling performance for the Li–S pouch cell with S/P‐TiO_2−*x*_ cathodes at 0.05C.

The CV of the first three cycles of the Li–S battery using the S/P‐TiO_2−*x*_ composite are shown in **Figure** **7**a. The CV curves of S/P‐TiO_2−*x*_ maintained a similar shape in the first three cycles. In the anodic branch (III), the anodic peak corresponds to the gradual transition of Li_2_S to LiPSs and finally to S_8_ during charging. During the first cycle of the S/P‐TiO_2−*x*_ cathode, the area of the anodic peak (*A*_3_) was almost equal to the area of the two cathodic peaks (*A*_1_ + *A*_2_), indicating that the electrical properties of the S/P‐TiO_2−*x*_ cathode have high reversibility and good capacity retention. The S/P‐TiO_2−*x*_ cathode was further used in Li–S pouch cells with high loading of S cathode (total sulfur mass of 49.6 mg, S loading ≈ 3.1 mg cm^−2^, electrolyte to sulfur = 6.25 mL g^−1^) (Figure 7b–d). The charge and discharge curves of the pouch at the 26th cycle are shown in Figure 7c, the charging and discharging platform can be clearly observed, which proves its good reaction kinetics. As shown in Figure 7d, a specific capacity of 639 mAh g^−1^ was achieved at 0.05C after 50 cycles, suggesting a decent cycle performance of the pouch cell.

## 3 Conclusions

We have proved that the well‐controlled and defective mesoporous P‐TiO_2−*x*_ microspheres as sulfur host material can significantly improve the coulombic efficiency and cycle stability of Li–S batteries. The ordered mesoporous P‐TiO_2−*x*_ microspheres with a shape similar to waxberry have radial mesoporous channels, oriented rutile crystallites, and a large number of stable defects. The 3D open‐pore mesoporous arrangement provides an effective transmission path, and the introduction of defects during P doping greatly improves ion diffusion kinetics. Therefore, S/P‐TiO_2−*x*_ cathode material achieves an excellent capacity of 1174.9 mAh g^−1^ at 0.2C, a coulombic efficiency of 99.7%, and a capacity retention rate (the average capacity attenuation is only 0.086% per cycle after 600 cycles at 1C). The amazing ability of mesoporous P‐TiO_2−*x*_ microspheres as the sulfur host material provides inspiration for the development of long‐cycle Li–S batteries.

## 4 Experimental Section

4.1

4.1.1

##### Materials Synthesis

Chemicals: TBOT (99%), Pluronic F127, tetrahydrofuran (THF, 99%), and sodium hypophosphite (NaH_2_PO_2_·H_2_O, 98%) were purchased from Sigma‐Aldrich. AcOH (99%), HCl (36 wt%), and NaBH_4_ (96%) were purchased from Sinopharm Chemical Reagent Co., Ltd.

##### Preparation of Rutile TiO_2_ Microspheres

Dissolve 1.6 g Pluronic F127, 2.0 mL AcOH, and 3.0 mL HCl in 30 mL THF and 3.0 mL TBOT and 0.20 mL H_2_O were added dropwise. The resulting golden yellow solution was dried at 45 °C for 24 h. Then the formed single micelle gel was heated in a 50 mL reaction kettle at 70 °C for 24 h.

##### Synthesis of Defective Mesoporous P‐TiO_2−*x*_ Microspheres

First, black TiO_2−*x*_ microspheres were prepared, evenly mixed TiO_2_ microspheres and NaBH_4_ (the mass ratio is 2:1) and heated them at 350 °C for 3 h under Ar atmosphere protection. Then washed them with 0.1 M HCl to remove excess sodium borohydride. Transfer the TiO_2−*x*_ powder (0.2 g) and NaH_2_PO_2_ (2.0 g) to a porcelain boat and perform phosphating treatment by heating at 300 °C for 2 h under argon protection. The resulting dark black powder was P‐TiO_2−*x*_.

##### Synthesis of S/P‐TiO_2−*x*_ and S/TiO_2_

Mix prepared P‐TiO_2−*x*_ (or TiO_2_) microsphere material with sulfur powder (mass ratio 1:3) and grind together. Then, the mixed materials were transferred to the inner lining of the reactor and sealed, heated to 155 °C in an Ar atmosphere, and kept for 8 h. After the reactor is naturally cooled to room temperature, S/P‐TiO_2−*x*_ (or S/TiO_2_) composite material is obtained.

##### Materials Characterization

The TEM images were collected on a 200 kV JEOL JEM‐2100 F combined. Take photographic images with a digital camera. X‐ray diffraction (XRD) using a laboratory‐based X‐ray source. All samples in XRD measurements were directly exposed to the air. The N_2_ adsorption isotherm was collected on the Micromeritics Tristar 3020 analyzer at 77 K. The Raman spectrum was recorded on a Horiba Scientific Raman spectrometer, and the excitation wavelength was generated by the Ar^+^ laser at room temperature was 514.5 nm. Collect defects by EPR spectrometer (FA‐200, JEOL). All calibrations refer to the C 1*s* peak of surface amorphous carbon.

##### Electrochemical Measurements

This article used composite sulfur material as the battery‐positive electrode, metallic lithium as the negative electrode, and Celgard 2400 as the separator. The electrolyte was 1 M lithium bistrifluoromethanesulfonimide dissolved in 1,3‐dioxolane (DOL) and ethylene glycol dimethyl ether (DME) (volume ratio 1:1) and added a mixed solution of 1 wt% LiNO_3_. Assembled the batteries (2032‐type coin cells) in an argon glove box.

##### Composite Positive Electrode

The composite material, conductive carbon black (Super P), and binder polyvinylidene fluoride) were uniformly dispersed in an appropriate amount of *N*‐methyl‐2‐pyrrolidone at a mass ratio of 8:1:1. Active material loading is about 1 mg cm^−2^. In the solvent, a uniform slurry was obtained. The slurry was uniformly coated on the carbon‐containing aluminum foil to obtain a working electrode.

##### Electrochemical Performance Test

Perform constant current charge and discharge, long cycle, and rate performance were tested on the Neware test system. 1.7–2.8 V was the voltage test window. The specific capacity value was calculated according to the mass of sulfur and the current ratio used. The cyclic voltammograms (CV) and electrochemical impedance spectroscopy (EIS) tests were carried out on the electrochemical workstation (CHI660E), and the EIS scanning frequency range was 100 kHz–0.01 Hz.

##### Pouch Cell

The preparation of the cathode of the pouch cell was the same as that of the coin cell, and the area of the cathode was 16 cm^−2^. The pouch cell was assembled in the glove box filled with Ar. The pouch cell was tested in the Neware test system at 0.05C within a voltage window of 1.7–2.8 V.

##### Symmetrical Battery

P‐TiO_2−*x*_ (or TiO_2_) was used as the working electrode, and carbon cloth was used as the counter electrode. A DOL/DME (volume ratio 1:1) solution containing 0.125 M Li_2_S_6_ was used as the electrolyte. The CV measurement was tested on the electrochemical workstation (CHI660E), the voltage range was −1 to 1 V, and the sweep speed was 5 mV s^−1^. The EIS measurement was also tested on the electrochemical workstation (CHI660E).

##### Li_2_S Deposition

Li_2_S_8_/TEGDME was used as the electrolyte. First, discharged the batteries at a constant current to 2.06 V, and then applied 0.01 V overpotential to drive the Li_2_S deposition.

## Conflict of Interest

The authors declare no conflict of interest.

## Supporting information

Supplementary Material

## Data Availability

The data that support the findings of this study are available from the corresponding author upon reasonable request.
