# Supplementary material for: Waxberry‐Shaped Ordered Mesoporous P‐TiO2−x Microspheres as High‐Performance Cathodes for Lithium–Sulfur Batteries
Source: Small Sci. 2022 Dec 1;3(1):2200032. doi: 10.1002/smsc.202200032 (PMC11936039; doi:10.1002/smsc.202200032)
Supplement: Supplementary file 1 — Supplementary Material [file SMSC-3-2200032-s001.pdf]

Supporting Information

Waxberry-Shaped Ordered Mesoporous P-TiO_{2-x} Microspheres as High-Performance Cathodes for Lithium–Sulfur Batteries

Wenna Zhang[a], Yuanmei Xu[a], Jiabing Liu[b], Yebao Li[a], Eser Metin Akinoglu[a], Yaojie Zhu[a], Yongguang Zhang[a.b], Xin Wang[a*], Zhongwei Chen[c*]*

[a] South China Academy of Advanced Optoelectronics, International Academy of Optoelectronics at Zhaoqing, South China Normal University, Guangdong, 510006, China

[b] School of Materials Science and Engineering, Hebei University of Technology, Tianjin, 300130, China

[c] Department of Chemical Engineering, University of Waterloo, Waterloo, ON, Canada, N2L 3G1

Corresponding Author

Zhongwei Chen: zhwchen@uwaterloo.ca
Xin Wang: wangxin@scnu.edu.cn
Yongguang Zhang: yongguangzhang@hebut.edu.cn

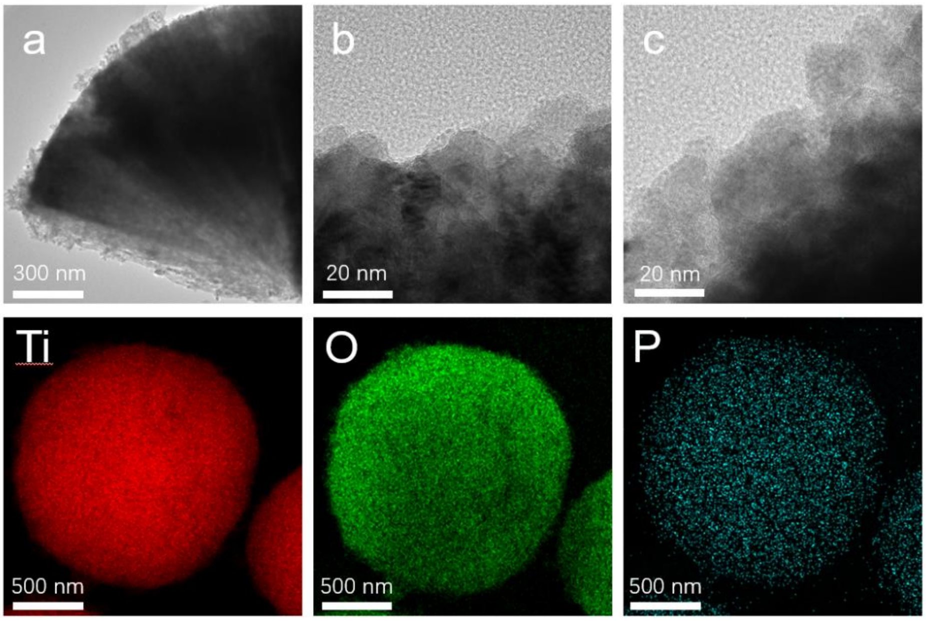

Figure S1. (a-c) TEM images and mapping images of P-TiO_{2-x} microspheres.

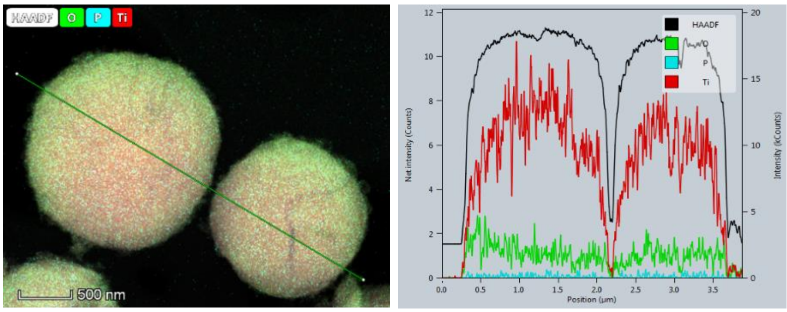

Figure S2. HAADF-STEM images of P-TiO_{2-x} microspheres.

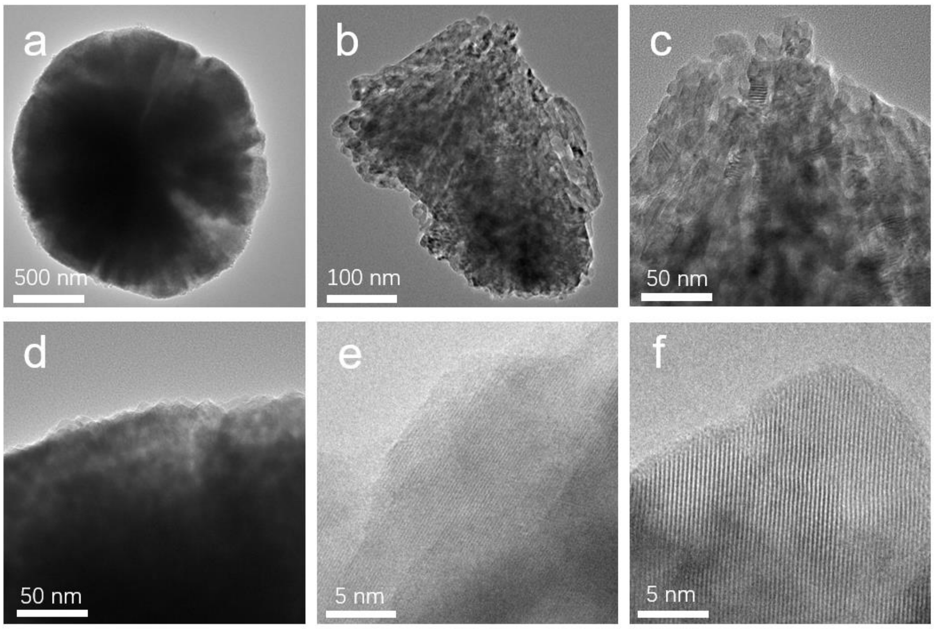

Figure S3. (a-f) TEM images of TiO_2 microspheres.

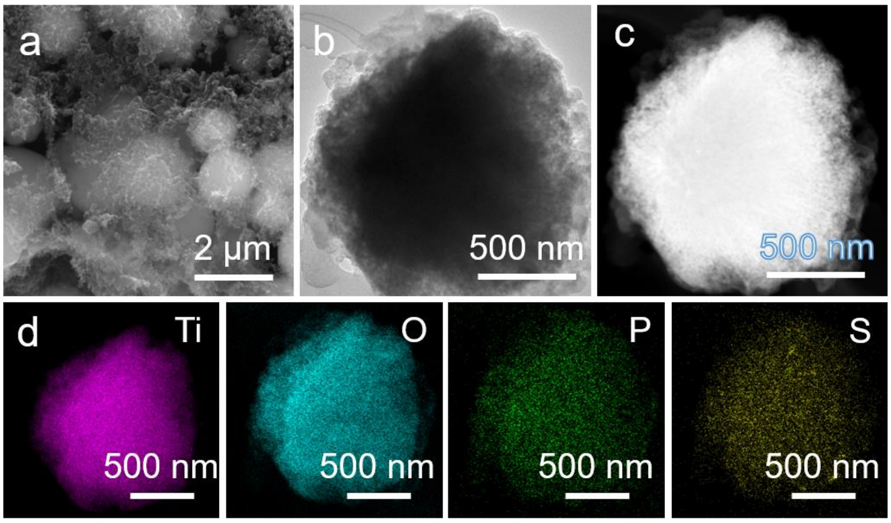

Figure S4. (a) SEM image of S/P-TiO_{2-x}, (b) TEM, (c) HAADF images and (d) Corresponding element mapping of S/P-TiO_{2-x}.

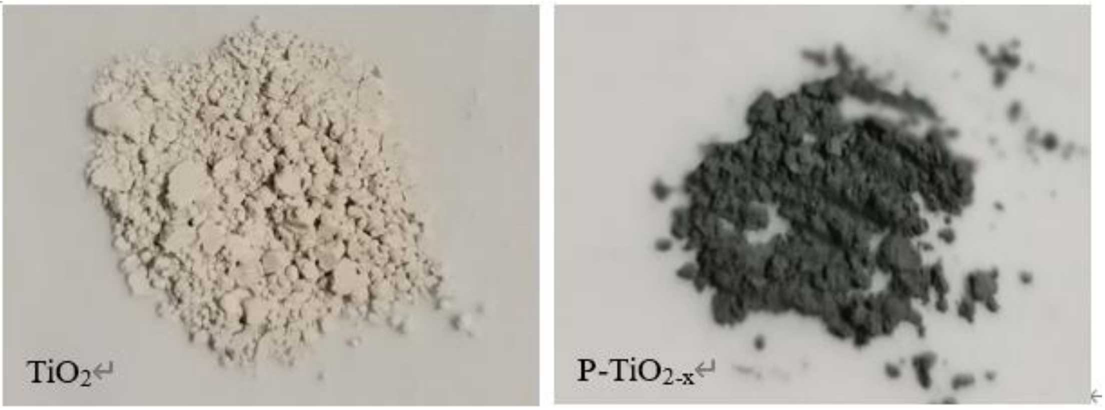

Figure S5. Different powders of TiO_2 materials.

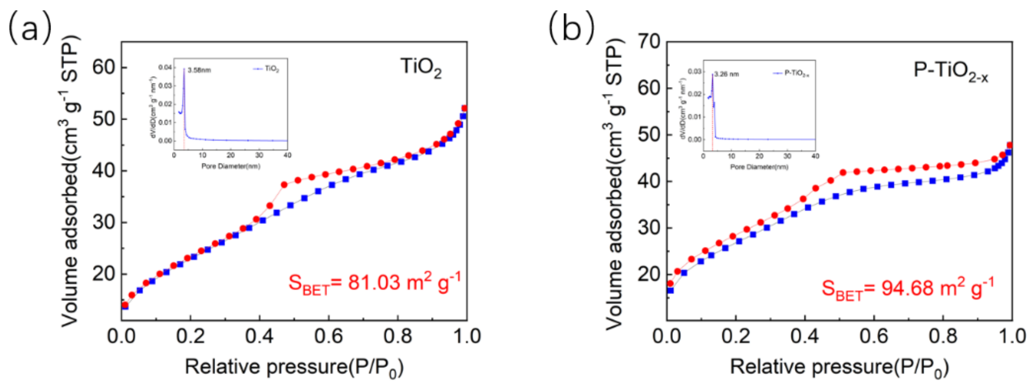

Figure S6. N_2 adsorption/desorption isotherms and corresponding pore size distribution of (a) TiO_2 and (b) P-TiO_{2-x}.

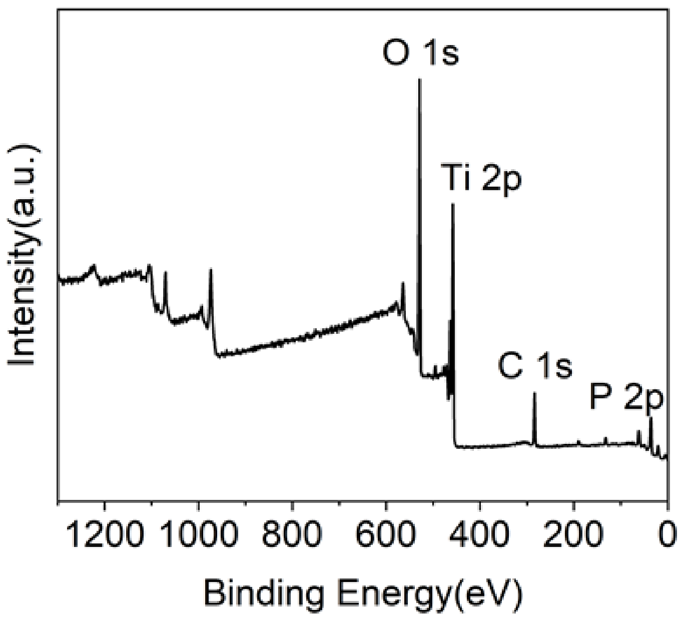

Figure S7. XPS survey of P-TiO_{2-x}.

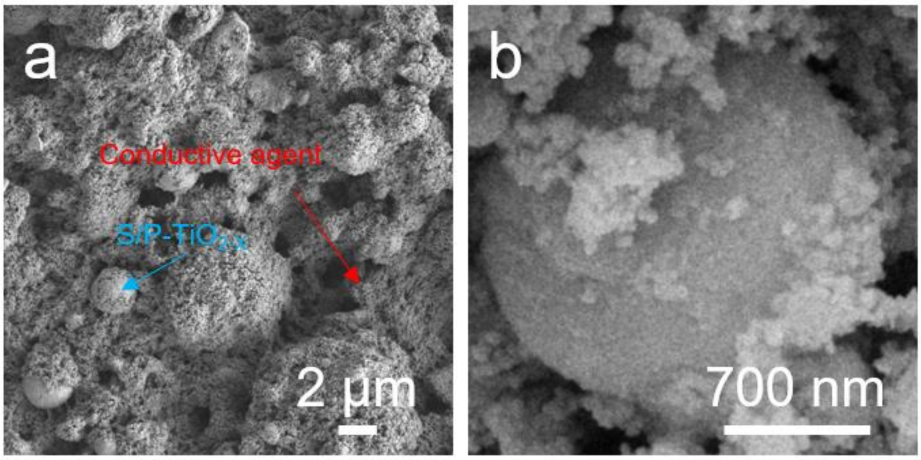

Figure S8. (a and b) SEM images of S/P-TiO_{2-x} cathode after cycling.

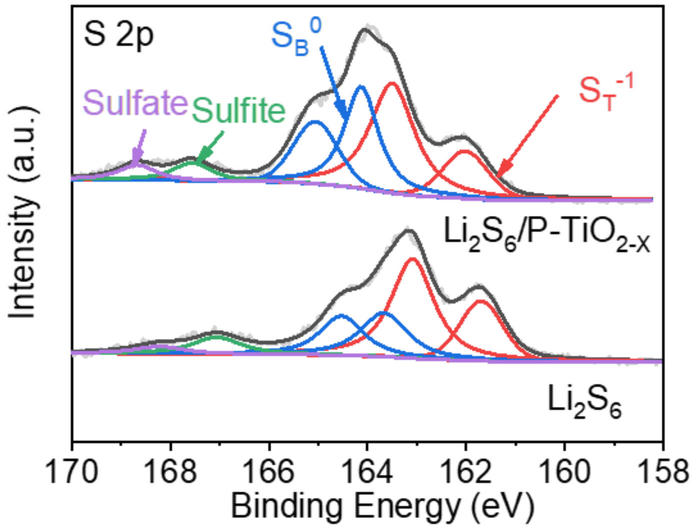

Figure S9. The XPS S 2p spectrum of Li_2S_6 and Li_2S_6/P-TiO_{2-x}.

Table S1. Atomic composition of P-TiO_{2-x} in XPS analysis.

Element	Ti	O	P	C
Composition	22.76	51.3	3.03	22.91

Table S2. Porous structure parameters of different samples.

	Surface area (m^2 g^{-1})	Pore volume (cm^3 g^{-1})
TiO_2	81.03	0.08
P-TiO_{2-x}	94.68	0.07

Table S3. The electrochemical performance of TiO_2-based cathodes.

Cathodes	Current (C)	Capacity retention (mAh g^{-1})	Cycle number	Ref.
$TiCeTiO_2$/SWCNT/S	1	666.5	200	[1]
S@void@TiO_2	1	580	400	[2]
TiO_2-B/S	0.2	572	100	[3]
TiO_2/C-S	0.5	780.6	100	[4]
S-CoS_2-TiO_2@C	1	736.5	300	[5]
S/P-TiO_{2-x}	**1**	**500.5**	**600**	**This work**

REFERENCES

(1) Geng, X. W.; Yi, R. W.; Lin, X. F.; Liu, C. G.; Sun, Y.; Zhao, Y. C.; Li, Y. Q.; Mitrovic, I.; Liu, R.; Yang, L.; Zhao, C. Z. A high conductive TiC-TiO_2/SWCNT/S composite with effective polysulfides adsorption for high performance Li-S batteries. *Journal of Alloys and Compounds* **2021,** 851, 156793

(2) Yu, Y.; Yan, M.; Dong, W.-D.; Wu, L.; Tian, Y.-W.; Deng, Z.; Chen, L.-H. Hasan, T.; Li, Y.; Su, B.-L. Optimizing inner voids in yolk-shell TiO_2 nanostructure for high-performance and ultralong-life lithium-sulfur batteries. *Chemical Engineering Journal* **2021,** 417, 129241.

(3) Dong, W. J.; Wang, D.; Li, X. Y.; Yao, Y.; Zhao, X.; Wang, Z.; Wang, H.-E.; Li, Y.; Chen, L. H.; Qian, D.; Su B.-L. Bronze TiO_2 as a cathode host for lithium-sulfur batteries. *Journal of Energy Chemistry* **2020,** 48, 259–266.

(4) Qi, C.; Li, H. L.; Wang, J.; Zhao, C .C.; Fu, C. M.; Wang, L. N.; Liu, T. X. Metal-organic framework derived porous carbon embedded with TiO_2 nanoparticles as cathode for advanced lithium-sulfur battery. *ChemElectroChem* **2021,** 8, 90–95.

(5) Li, D. M.; Li, H. T.; Zheng, S. M.; Gao, N.; Li, S.; Liu, J.; Hou, L. L.; Liu, J. C.; Miao, B. B.; Bai, J.; Cui, Z. M.; Wang, N.; Wang, B.; Zhao, Y. CoS_2-TiO_2@C core-shell fibers as cathode host material for high-performance lithium-sulfur batteries. *Journal of Colloid and Interface Science* **2022,** 607, 655–661.
